# Estimation of *Trypanosoma cruzi* infection in the main vector *Triatoma infestans*: accounting for imperfect detection using site-occupancy models

**DOI:** 10.1186/s13071-025-06693-3

**Published:** 2025-02-18

**Authors:** Marta Victoria Cardinal, Gustavo Fabián Enriquez, María Sol Gaspe, María del Pilar Fernández, Victoria Capello, Ricardo Esteban Gürtler

**Affiliations:** 1 Facultad de Ciencias Exactas y Naturales, Laboratorio de Eco-Epidemiología, Universidad de Buenos Aires, Int Güiraldes 2160, 2do piso, Ciudad Universitaria, C1428EHA Buenos Aires, Argentina; 2 CONICET-Universidad de Buenos Aires, Instituto de Ecología, Genética y Evolución de Buenos Aires (IEGEBA), Ciudad Universitaria, Buenos Aires, Argentina; 3 Paul G. Allen School for Global Animal Health, Washington State University, Pullman, WA USA

**Keywords:** *Trypanosoma cruzi*, *Triatoma infestans*, Triatomine, Chagas disease, Diagnosis, PCR, Imperfect detection, Occupancy models

## Abstract

**Background:**

Vector infection prevalence is a key component of vectorial capacity and transmission risk. Optical microscopy observation (OM) of fecal drops has been the classic method for detecting *Trypanosoma cruzi* infection in triatomine bugs until the advent of polymerase chain reaction (PCR)-based techniques. However, agreement among OM- and PCR-based techniques has been highly heterogeneous.

**Methods:**

We used hierarchical site-occupancy models accounting for imperfect detection to estimate method-specific detection probabilities of *T. cruzi* infection in field-collected *Triatoma infestans* and to assess whether *T. cruzi* infection varied with triatomine developmental stage and collection ecotope. We also performed a scoping review of the literature on comparisons between OM and PCR for *T. cruzi* infection diagnosis in triatomines. Triatomines were collected before vector control interventions in Pampa del Indio houses (Argentine Chaco) and examined by OM. We amplified the variable regions of the kinetoplastid minicircle genome (vkDNA-PCR) in DNA extracted from the rectal ampoules of 64 OM-positive and 65 OM-negative *T. infestans.*

**Results:**

vkDNA-PCR detected *T. cruzi* infection in 59 (92.2%) OM-positive bugs and in 19 (29.2%) OM-negative triatomines in blind tests. The overall prevalence of infection, as determined by a positive test result by either vkDNA-PCR or OM, was 64.3% [95% confidence interval (95% CI) 55.8–72.1%]. Detection probability of *T. cruzi* infection by vkDNA-PCR (92%, 95% CI 83–97%) was substantially higher than for OM (76%, 95% CI 65–84%). Infection was minimal (26.2%) in peridomestic nymphs and maximal in domestic adult triatomines (81.7%). In the literature review encompassing 26 triatomine species from 11 countries, inter-method agreement ranged from 28.6% to 100%. The lowest agreement was observed in *Rhodnius sp.* and *Panstrongylus lutzi* and the highest among *Triatoma sp.*, with wide variability in the protocols and outcomes of molecular diagnosis in comparison with OM.

**Conclusions:**

Our study provides a synthesis on the different sources (both biological and technical) of variation of the outcomes of OM- and PCR-based diagnosis of *T. cruzi* infection in triatomines and identifies new research needs for diagnostic improvement.

**Graphical Abstract:**

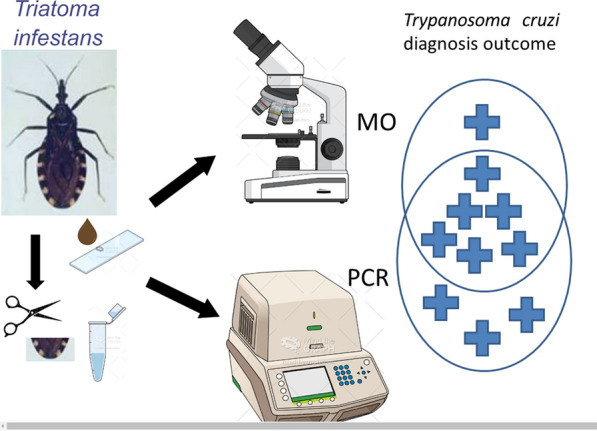

**Supplementary Information:**

The online version contains supplementary material available at 10.1186/s13071-025-06693-3.

## Background

*Trypanosoma cruzi*, the etiological agent of Chagas disease, is a multi-host protozoan transmitted by different routes: vector-borne, vertical, oral, and by transfusion or transplantation of infected organs [55]. Although more than 70 triatomine species (Hemiptera: Reduviidae: Triatominae) have been found naturally infected with *T. cruzi*, three genera (*Triatoma, Rhodnius*, and *Panstrongylus*) concentrate the most important species for human and veterinary health [2, 8, 15, 46].

Optical microscopy observation (OM) of fecal drops has been the standard method for detecting *T. cruzi* infection in triatomine bugs. Its strengths include the speed of results, easy implementation under field conditions, compatibility with keeping the insect alive, and low cost [27]. Among its limitations, OM is a labor-intensive method and the outcomes heavily depend on technician skills, it has low specificity in the presence of other trypanosomatids such as *Trypanosoma rangeli* [14] and *Blastocrithidia triatomae* [13, 44], widely variable sensitivity, especially among the smaller nymphal stages, and potential biohazard risks linked to the manipulation of infectious samples.

With the advent of polymerase chain reaction (PCR)-based techniques, *T. cruzi* infection diagnosis has increasingly relied on them given its high specificity and sensitivity, in spite of its higher cost. PCR-based methods may give false negative results if DNA is contaminated with PCR inhibitors [16], or false positive results due to contaminations (cross-contamination of samples or contamination with target DNA amplicons).

Vector-borne *T. cruzi* transmission in the domestic environment depends on the occurrence and abundance of mammalian hosts acting as parasite sources, triatomine abundance, prevalence of bug infection, and vector–host contact [6]. Thus, assessing the occurrence and prevalence of *T. cruzi* infection in the vectors is a crucial component of transmission risk. Parasite-based indices can help assessing the progress toward the elimination of intradomiciliary vector-borne *T. cruzi* transmission, one of the goals set for Chagas disease by the World Health Organization in the 2030 roadmap for the elimination of Neglected Tropical Diseases [54]. However, imperfect detection of *T. cruzi* hampers our ability to estimate the occurrence of infection and vector-borne transmission risks.

Imperfect detection of the presence of a given species in a given site is a classic problem in ecological research [30], including the occurrence of triatomines and *T. cruzi* infection [1, 35]. The main consequence of imperfect detection is that the observed “naïve” prevalence most likely underestimates the true prevalence. Minuzzi-Souza et al. [35] evaluated the detection probability of five diagnostic methods of *T. cruzi* in six triatomine species barring *Triatoma infestans*; they showed that OM missed between 50% and 75% of the infected triatomines, whereas quantitative polymerase chain reaction (qPCR) achieved > 99% sensitivity and specificity. Discrepancies among diagnostic methods varied widely among triatomines species, with *Triatoma sordida* exhibiting the smallest difference between microscopic and molecular outcomes. No such study has been performed for *T. infestans*, historically the main domestic vector of human infection with *T. cruzi* and possibly the most competent vector species among the Triatominae [37].

Herein we address the imperfect detection of *T. cruzi* infection in triatomines. By examining the same *T. infestans* individuals by OM and PCR, the probability of detecting *T. cruzi* can be estimated and a corrected estimate of infection prevalence computed. We used hierarchical site-occupancy models accounting for imperfect detection to estimate method-specific detection probabilities of *T. cruzi* infection in field-collected *T. infestans* and to assess whether *T. cruzi* infection varied with triatomine developmental stage (nymphs versus adults) and collection ecotope (domestic versus peridomestic). Secondly, we performed a scoping review of the literature on comparisons between OM and PCR for diagnosis of *T. cruzi* infection in triatomines to assess their inter-method agreement according to species and test protocols.

## Methods

### Triatomine collection

Field work took place in a section of Pampa del Indio municipality (Chaco Province, Argentina), as part of a longitudinal research and control program on the eco-epidemiology of Chagas disease [22]. The study area (denominated area 1) was described elsewhere [21]. Triatomines were collected during the baseline (pre-intervention) survey of house infestation (September–October 2007) and kept in labeled plastic bags according to collection site by house, and then taken to the field laboratory where they were identified to species and counted by sex and stage. All triatomines were kept alive and taken to the insectary at the Faculty of Natural and Exact Sciences in Buenos Aires for further processing.

*Triatoma infestans* was collected by timed-manual searches in 41.1% of the 313 rural houses of area 1 [22]. We have previously reported that the observed (i.e., naïve) prevalence of infection with *T. cruzi* in *T. infestans* was 27.2% (95% CI 25.3–29.3%) as determined by OM, whereas the overall seroprevalence of *T. cruzi* infection was 26.0% in domestic dogs; 28.7% in domestic cats [11], and 39.8% in the resident human population [12].

### Study design

All live third-instar nymphs and later stages of *T. infestans* were examined for *T. cruzi* infection by direct microscopy within 20 days of collection [11]. Following a standardized protocol, first- and second-instar nymphs were occasionally collected but were not examined for infection given the difficulty to manipulate small instars and their nil or very low prevalence of infection. A small fecal drop obtained by abdominal compression was mixed with one drop of saline solution on 22 × 22 mm slides and then it was thoroughly examined for trypanosomes at 400× by trained members of the research group. Insects were thereafter frozen at −20 °C. To compare the outcomes of OM and PCR diagnosis, a convenience sample (*n* = 129) of frozen microscopy-positive and microscopy-negative triatomines was tested by molecular methods. The examined insects were collected in eight houses from four rural villages.

### Molecular diagnosis

The study insects were dissected to extract their rectal ampoules, which were mixed with 50 µl of sterile water and boiled for 10 min. DNA was extracted from an aliquot of 25 µl of rectal ampoule lysate using DNAzol (Invitrogen, Carlsbad, CA, USA) following manufacturer instructions. We amplified a fragment of 330 bp from the variable region of minicircles of the kinetoplastid genome (vkDNA) as described [33] and used Taq Platinum to enhance sensitivity.

### Data analysis

We estimated the probability of detecting a *T. cruzi* infection by OM or PCR using single-season occupancy models implemented in the software PRESENCE 2.13.47 and the hierarchical-modeling approach described by Minuzzi-Souza et al. [35]. For this purpose we compiled a dataset (Additional File 2, Table S2) with 129 *T. infestans* examined for *T. cruzi* infection both by OM and vkPCR. We examined whether the occurrence of infection varied according to bug stage (nymphs versus adults) and collection ecotope (domiciles versus peridomestic habitats). We constructed five models that expressed different a priori hypotheses, where *Ψ* is the occupancy probability (i.e., probability of *T. cruzi* infection) and *P* the detection probability:

(i) *Ψ* (.), *P*(.): uniform detection and uniform infection probabilities.
(ii) *Ψ* (.), *P*(method): detection probability varying with the method, uniform infection probability.
(iii) *Ψ* (adult), *P*(method): detection probability varying with the method, infection probability varying with triatomine stage.
(iv) *Ψ* (domicile), *P*(method): detection probability varying with the method, infection probability varying with triatomine collection ecotope.
(v) *Ψ* (adult + domicile), *P*(method): method-dependent detection and infection occurrence varying with triatomine stage and collection ecotope.

Following the nomenclature employed in occupancy models, within brackets we denoted the covariates considered for each probability. The null model, with no covariates for occupancy and uniform detection probability, was denoted *Ψ*(.)*P*(.), where “.” denotes no covariate. We used Akaike’s information criterion (AIC) for model selection; hypotheses represented by models with lower AIC values are better supported by the data.

We illustrate the rationale of occupancy models using a hypothetical example in which the goal is to determine whether *T. cruzi* infection (occupancy, denoted *Ψ* in the abovementioned models) varies between two triatomine species (such as *T. infestans* and *T. sordida,*, which exhibit different domiciliation and infestation patterns in the Argentine Chaco and feed on different hosts, leading to *T. sordida* being rarely infected), and whether the detection probability of *T. cruzi* infection (denoted *P* in the abovementioned models) varies between two different PCR-based diagnosis protocols (protocol_1_ versus protocol_2_). The occupancy model creates two linked logit models: one for occupancy probability (*Ψ*) and one for detection probability (*P*), with *Ψ* being conditional on *P*. In this example the occupancy probability model (*Ψ*) estimates the probability of infection in a bug on the basis of species. The model first estimates logit coefficients, which are values on the log-odds scale, for each species or detection method relative to a baseline. To obtain interpretable probabilities, these logit values are back-transformed using the inverse logit function. For example, suppose *T. sordida* is the reference species, and the model provides an intercept of −0.8 for *T. sordida*, along with a coefficient of 1.1 for *T. infestans*. This coefficient indicates that *T. infestans* has a higher likelihood of infection than *T. sordida*. To calculate the probability of infection for each species, we apply the inverse logit transformation to the logit values 1/(1 + e^−Logit^). For *T. sordida*, the probability is calculated as 1/(1 + e^0.8^) ≈ 0.31, or 31%. For *T. infestans*, the logit is −0.8 + 1.1 = 0.3, so the probability is 1/(1 + e^−0.3^) ≈ 0.57, or 57%. This difference in back-transformed probabilities shows that *T. infestans* has a higher likelihood of infection than *T. sordida*. Similarly, if the detection probability model (*P*) for detection method protocol_2_ has a coefficient of 1.2 relative to protocol_1_, this back-transformed probability would show protocol_2_’s higher effectiveness at detecting infection.

The two models work together by incorporating detection history across multiple observations to jointly estimate *Ψ* and *P*. For example, if a bug has no detections over repeated tests, the model considers two possibilities: the bug is uninfected (true absence), represented by 1 − *Ψ*, or the bug is infected, but detection failed each time (false absence), represented by *Ψ* × (1 − *P*) for each observation. The model uses this likelihood function to combine observed detections and non-detections, thus linking occupancy and detection estimates. This joint estimation ensures that the probability of infection is adjusted for imperfect detection, resulting in an accurate estimate of the true infection probability in the bug population.

For the literature review, we retrieved previous studies that reported OM- and PCR-based diagnosis of *T. cruzi* infection in triatomines (natural or artificial infections) and excluded comparisons between OM and PCR for diagnosis of *T. cruzi* infection in mammalian hosts or tissues. Searches were performed in Pubmed, Google Scholar, BibTri, and Scielo from 1992 until June 2024, including literature published in English and Spanish. Searches were done employing the terms: “trypanosoma cruzi + PCR” or “trypanosoma cruzi + Triatoma/ Panstrongylus/ Rhodnius/ Mepraia”. The studies retrieved were then selected on the basis of the simultaneous use of PCR and OM for *T. cruzi* infection diagnosis in triatomines. We excluded: (i) reports of PCR and OM diagnosis in only one triatomine; (ii) reports pooling the outcomes for different triatomine species; (iii) reviews with no primary data; and (iv) unpublished thesis dissertations.

We calculated concordance between techniques as the percentage of samples positive and negative by both techniques [7] and the Kappa index using Epitools [45]. Examined insects were classified according to collection site in (peri)domestic (i.e., triatomines collected in domestic or peridomestic sites) and sylvatic.

## Results

**Fig.1:**
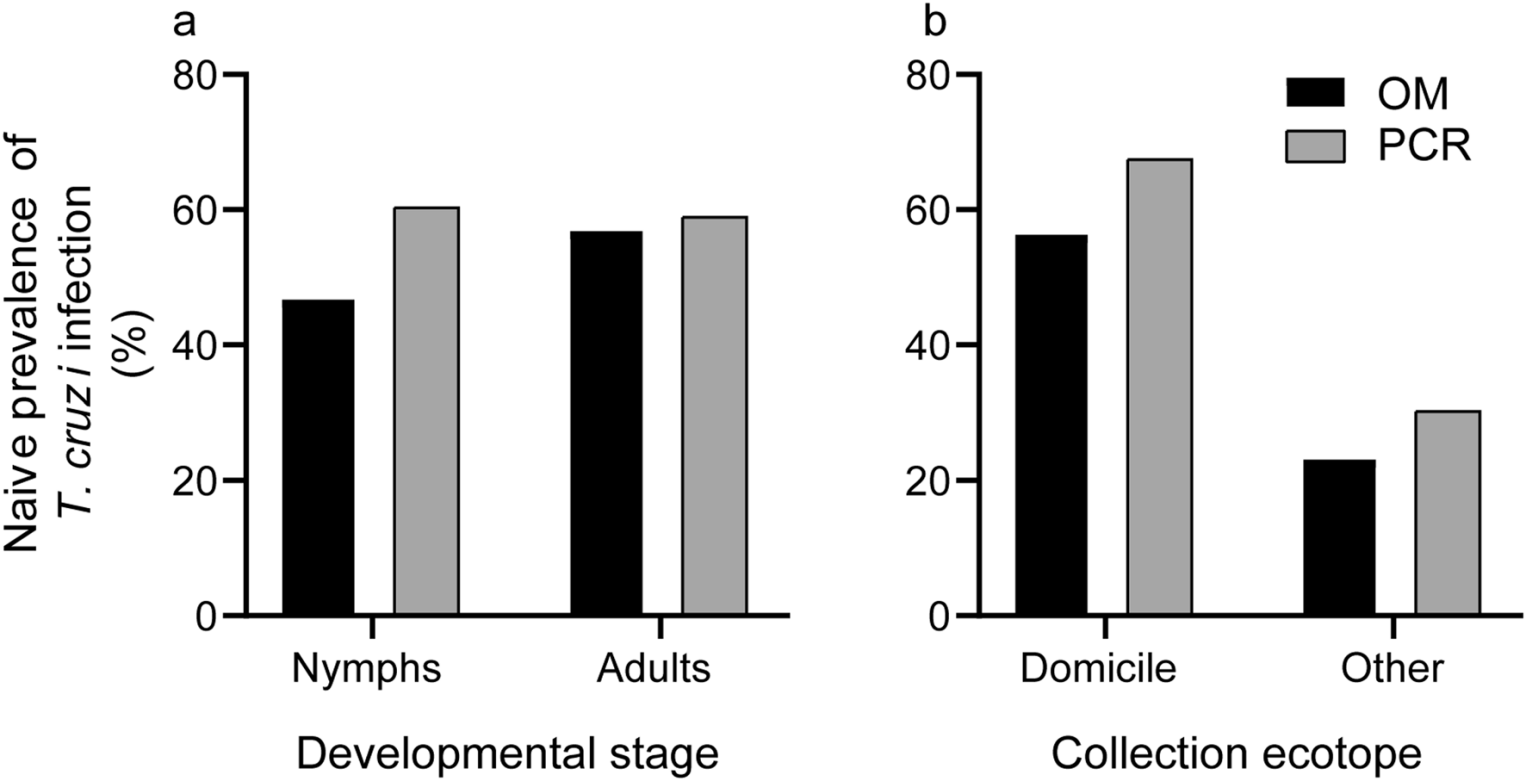
Naïve prevalence of *Trypanosoma cruzi* infection by optical microscopy (OM) and PCR in *Triatoma infestans* according to **a** developmental stage and **b** collection ecotope (other includes chicken coop, kitchen, and wood pile)

A total of 129 *T. infestans* examined microscopically (64 OM-positive and 65 OM-negative) were subsequently tested blindly by vkDNA-PCR. Examined triatomines were mainly collected from domiciles (80%); the remainder was collected in chicken coops (19%), and in a kitchen and a wood pile (1%) from eight houses. Co-positivity occurred in 59 (45.7%) insects; co-negativity in 46 (35.7%); 5 (3.9%) were OM-positive and vkDNA-PCR-negative, and 19 (14.7%) were microscope-negative and vkDNA-PCR-positive. Considering an insect infected on the basis of vkDNA-PCR and/or OM (i.e., by either method), the overall prevalence of infection was 64.3% [95% confidence interval (95% CI) 55.8–72.1%]. OM-positivity occurred in 71.1% of these insects, and vkDNA-PCR-positivity in 94.0%. vkDNA-PCR detected *T. cruzi* infection in 92.2% (*n* = 64) and 29.2% (*n* = 65) of OM-positive and OM-negative triatomines, respectively. The naïve prevalence of infection by OM was higher in domestic (56.3%, *n* = 103) than in peridomestic (23.1%, *n* = 26) bugs (Fig. 1B) and was slightly higher in adults (56.8%, *n* = 37) than in nymphs (46.7%, *n* = 92) (Fig. 1A).

To account for imperfect detection, we first run the simplest model considering uniform *T. cruzi* infection probability and uniform detection probabilities across triatomines and techniques. This model had the least support (Table 1). The best model accounted for different detection probabilities for OM and vkPCR and variation in infection by collection ecotope. The support for the most complex model (in which infection status also varied with triatomine stage) differed little from the former model (ΔAIC < 0.6); therefore, these last two models were considered top models (Table 1). The detection probability of vkDNA-PCR (92%, 95% CI 83–97%) was higher than for OM (76%, 95% CI 65–84%); hence, OM missed almost 1 of every 4 infected triatomines collected before control interventions. Infection estimates ranged from 26.2% to 81.7% according to developmental stage and collection ecotope and were higher in adults (versus nymphs) and in triatomines collected in domiciles (versus peridomiciles). Parameter estimates and 95% CIs are presented in Table 2.

**Table 1:** Models of the occurrence and detection of *Trypanosoma cruzi* infection in *Triatoma infestans* collected before vector control interventions in Pampa del Indio, 2007

Model	AIC	ΔAIC	AIC weight	Model likelihood	*K*	−2 × Log-likelihood
*Ψ* (domicile.), *P*(method)	285.02	0	0.5714	1	4	277.02
*Ψ*(adult + domicile), *P*(method)	285.6	0.58	0.4276	0.7483	5	275.6
*Ψ* (.), *P*(method)	298.46	13.44	0.0007	0.0012	3	292.46
*Ψ* (adult), *P*(method)	300.23	15.21	0.0003	0.0005	4	292.23
*Ψ* (.), *P*(.)	305.17	20.15	0	0	2	301.17

The best supported model according to AIC is shown in the first row

*ΔAIC* delta AIC represents the variation of AIC relative to the best model

*k* number of parameters in the model

*Ψ* is the occupancy probability (i.e., probability of *T. cruzi* infection) and *P* the detection probability

**Table 2:** Back-transformed numerical estimates from logit to the probability scale

Model/parameter	Estimate (95% CI)
*Ψ* (domicile), *P* (method)	
Domicile	0.74 (0.64–0.82)
Peridomicile	0.31 (0.16–0.51)
OM	0.75 (0.65–0.84)
PCR	0.92 (0.83–0.97)
*Ψ* (adult + domicile), *P* (method)	
Adult + domicile	0.82 (0.64–0.92)
Adult + peridomicile	0.38 (0.19–0.63)
Nymphs + domicile	0.72 (0.60–0.80)
Nymphs + peridomicile	0.26 (0.12–0.48)
OM	0.75 (0.65–0.84)
PCR	0.92 (0.83–0.97)

The 19 OM-false negative outcomes occurred in three domiciles and one chicken coop from four houses. The three domiciles harbored other infected triatomines as determined by both OM and PCR, whereas the remaining two PCR-positive insects (a female and a fifth-instar nymph) from the chicken coop were the only ones observed positive in this site. The few OM-positive, vkPCR-negative insects found were probably explained by the presence of PCR inhibitors. The finding of other triatomine bugs (range 4–17) positive by OM and vkPCR in the same collection sites as the putatively inhibited samples further supports the occurrence of *T. cruzi* infection at the collection site level. These results agree with the high aggregation of *T. cruzi* infection at the collection site and household levels.

**Fig. 2:**
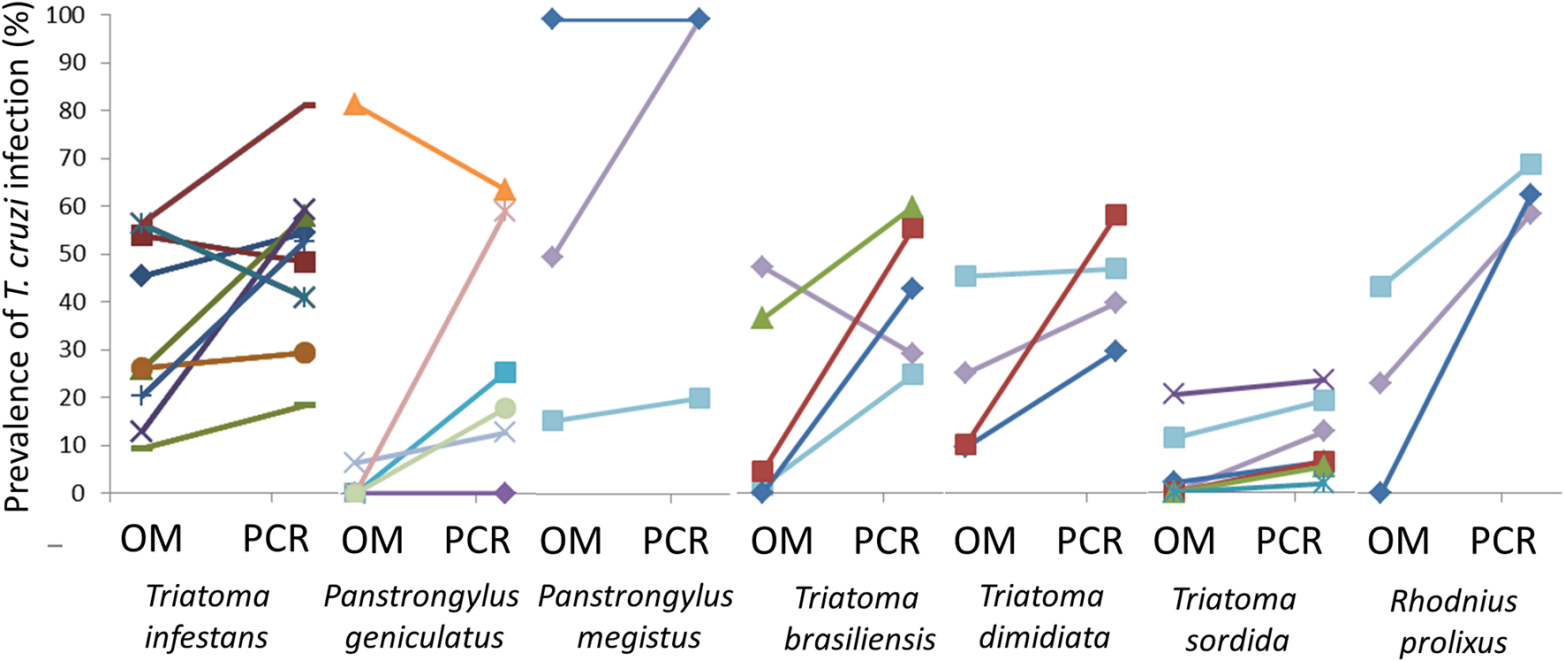
Outcome of *Trypanosoma cruzi* infection by optical microscopy (OM) and PCR across the main triatomine species

The published reports comparing OM and PCR diagnosis of *T. cruzi* infection in triatomines show that concordance between techniques ranged from 47.2% to 100% and differed widely across the 26 triatomine species considered (Fig. 2, Additional File 1, Table S1). Regarding *T. infestans*, concordance between OM and PCR outcomes was high and usually ranged between 85.3% and 95.4%. The two studies with intermediate concordance (53.7–58.3%) involved triatomines used in xenodiagnosis; in one of them, triatomines were individually examined by OM and tested by PCR in pools of ten insects. We found no report of *T. rangeli* naturally infecting *T. infestans* or other species of the Triatomini tribe, whereas *T. rangeli* was widespread among *Rhodnius spp*. This review shows a plethora of different protocols employed for molecular diagnosis: at least eight different DNA extraction methods, five different amplification targets, and even different primer pairs were employed for the same target (Additional File 1, Table S1).

We compared our results with those from a similar study that also employed Taq Platinum and the same molecular diagnosis protocols, while using fecal lysates instead of rectal ampoules, for *T. infestans* collected pre- and post-intervention in Santiago del Estero, Argentina [33]. The fraction of OM-positive triatomines detected by vkDNA-PCR in Pampa del Indio was indistinguishable from the equivalent estimate in Santiago del Estero (91%) (Fisher’s exact test, *df* = 1, *P* = 1.0). By contrast, the fraction of OM-negative, vkDNA-PCR-positive *T. infestans* in Pampa del Indio was nearly fourfold higher than in Santiago del Estero (Fisher’s exact test, *df* = 1, *P* < 0.001). In Pampa del Indio, the overall infection prevalence was 27.2%, whereas in Santiago del Estero it ranged from 4.5% to 13.2% in two study areas [10].

## Discussion

Our results confirm the occurrence of imperfect detection of *T. cruzi* in field-collected *T. infestans*, with a substantial fraction of infected bugs missed by OM (25%), and support the need of estimating appropriate correction factors. The literature review supports that imperfect detection of *T. cruzi* infection can be generalized to all triatomine species examined thus far. Accounting for imperfect detection is needed to estimate actual transmission risks across collection ecotopes and epidemiological contexts.

The challenge of estimating the prevalence of a pathogen can be paralleled to the estimation of population abundance or density. Sometimes the exact estimate is needed, but in most cases it is not or it is too costly to obtain, and a consistent population index is sufficient [48]. The imperfect detection of *T. cruzi* infection by OM should be better considered an index of the natural infection prevalence, and used for comparative purposes and trend detection when protocols are optimized and held constant over time and space. Its choice is justified by lower costs and equipment requirements that for molecular-based diagnosis. Instead of only focusing on the amount of discrepancy, it is critical to evaluate its sources of variation (e.g., stage, ecotope, intervention phase).

In natural xenodiagnosis of dogs and cats, vkDNA-PCR revealed *T. cruzi* infection in 13% of OM-negative fourth-instar nymphs of *T. infestans* (*n* = 94), whereas false-negative results were concentrated on the most infectious hosts [18]. In artificial xenodiagnosis of human blood samples using fourth-instar nymphs of *T. infestans*, we also found 13% of vkDNA-PCR-positive, OM-negative (*n* = 527) triatomines [31], suggesting a constant detection error under the same protocols. In the current study, the fraction of vkPCR-positive and OM-negative insects was nearly twofold higher than in those xenodiagnostic studies using a defined triatomine stage. Additionally, the fraction of vkPCR-positive and OM-negative insects was also higher than in the Santiago del Estero study [33]. Whether this difference is due to the sources of DNA used for PCR (fecal lysates versus rectal ampoules) or to dissimilar epidemiological settings and triatomine collection sites remains to be evaluated.

Nearly 25% of the triatomines observed infected by either technique were missed by OM in the current study. Using the percentage of false-negative triatomines by OM (14.7%) herein estimated, we computed adjusted prevalence rates of domestic triatomine infection by operational area of Pampa del Indio from data in Gürtler et al. [23] (Supplementary Table 1). The naïve (i.e., observed) prevalence of *T. cruzi* infection in domestic *T. infestans* from operational areas I–IV was 35.6%, 45.2%, 26.7%, and 12.2%, respectively, whereas the adjusted prevalence was 45.0%, 53.3%, 37.4%, and 25.1%, respectively. Given that at the program endpoint assessment all triatomines were tested by PCR, in this case accounting for imperfect detection by OM during the initial phases of the intervention program returns a stronger effect of control actions on triatomine infection rates. Further studies assessing *T. cruzi* infection simultaneously by OM and PCR in triatomines collected after sustained control are needed to evaluate whether the detection probabilities are modified by the intervention phase (i.e., attack versus surveillance phase). PCR also missed nearly a 10% of OM-infected triatomines under our test conditions. Hence, imperfect detection by OM and PCR may underestimate the actual risks of individual host exposure to largely different degrees.

The probabilities of false-negative and false-positive results of a test with given specificity and sensitivity vary with the underlying true prevalence of the pathogen ([19]: *P*. 7). This implies that different correction factors are needed depending on the intervention phase when triatomines were collected. Ideally, the reviewed studies in Addtitional File 1, Table S1 should have been classified according to whether they pertained to high- or low-risk transmission scenarios to infer the probability of *T. cruzi* infection and the resulting probabilities of false-positive and false-negative results. Unfortunately, most reports lacked this key piece of information.

The literature review confirms the widely accepted notion that PCR is substantially more sensitive than OM for detecting *T. cruzi* infection, but there were widely different results among triatomine species (Fig. 2, Additional File 1, Table S1). The greatest disagreement between OM and PCR outcomes occurred in *Rhodnius pictipes* and *Panstrongylus lutzi* and the smallest one among *Triatoma* species. The absence of *T. rangeli* infections among species of the tribe Triatomini [51, 52] and the very low prevalence of *B. triatominae* (which can be differentiated by trained microscopists) [34] probably explain the higher inter-method concordance. For *T. infestans*, pooling triatomine samples apparently amplified the discrepancy between OM and PCR outcomes, and targeting kDNA was judged to be the best option. The performance of microscopical observation depends crucially on the effort deployed (cover/slide size, search time, and magnification). However, wide variations on these key aspects were also observed: some protocols only invested 5 min per sample whereas others searched all fresh slide fields. Increasing the number of fresh slide fields examined could help increase the sensitivity of OM [35]. It is worth noting that there is no gold standard molecular method for diagnosis of *T. cruzi* in triatomines thus far. Large variation in the detectability of *T. cruzi* infection occurred among nymphal stages [4, 27]. Whether the observed variability can be explained by differential parasite load according to triatomine species and stage remains to be determined [17]. Indeed, median parasite loads substantially differed among several triatomine species [36] though no significant differences among developmental stages were observed in *Rhodnius prolixus* [50]. If reinfections contribute to higher parasite loads, domiciliated triatomine species collected in active transmission scenarios may exhibit higher parasite loads than sylvatic, intrusive triatomine species. This may also contribute to the higher agreement between OM and PCR diagnosis observed for *T. infestans*. The reviewed studies also differed widely in the number of tested triatomines, collection sites, and whether they tested natural or experimental infections. For nearly a half (14 of 26) of the reviewed species, only one study assessed the relative outcomes of PCR and OM. More studies are needed to assess the performance of these diagnostic methods for the understudied triatomine species.

An overwhelming amount of PCR-based assays have been used for the molecular diagnosis of *T. cruzi* infection (Bautista-López and Ndao [3], for a review of diagnosis in humans and mammalian hosts), and for *T. cruzi* discrete typing unit (DTU) identification [53]. The study protocols for Triatominae differed in the amplification targets, PCR primers and cycling conditions employed, DNA extraction methods, DNA volume employed for amplification, source of parasite DNA (feces, rectal ampoule, intestinal, and stomach contents), type of polymerase employed, and preservation methods. These multiple differences hamper any direct comparison of study protocols, which involved different manipulation costs and need of technical expertise. A recent study highlighted the influence of DNA extraction methods on the ability to detect *T. cruzi* in human samples. In the present study and in previous ones [33, 32, 44], we have used Taq Platinum (Invitrogen) to prevent unspecific amplification and achieve enhanced sensitivity. Inhibition of PCR is a recognized source of false-negative PCR outcomes in OM-positive triatomines [43]. Within the distribution area of *T. rangeli* and particularly when dealing with *Rhodnius* sp., false-positive OM results can be expected [17]. Different amplification targets (nuclear DNA or kDNA) and sets of primers have also been pointed as sources of variation in PCR sensitivity [5]. Quantitative PCR assays outperformed conventional PCR in the detection of *T. cruzi* in triatomines and achieved maximum predictive values [35, 36]. However, its elevated costs make it unaffordable for vector surveillance systems. A standardized, cheap-as-possible, molecular diagnosis protocol of *T. cruzi* infection in triatomines may be instrumental to assess the status of transmission. Promising results of loop-mediated isothermal amplification (LAMP) have recently been reported [28]. Without the need of prior DNA extraction, LAMP could be implemented at the field sites as part of regular vector surveillance operations given that no expensive equipment is required, with the advantage of obtaining quick results.

One of the main limitations of our study relates to the use of convenience samples enriched in domestic triatomines, which did not allow us to compare the relative performance of PCR and OM across a wider range of bug ecotopes, which usually differ in triatomine infection prevalence. Domestic *T. infestans* usually exhibit higher OM-prevalence than triatomines collected in nearby peridomestic sites (i.e., kitchens, storerooms, mud ovens). If the prevalence of bug infection was positively associated with parasite burden in bug feces, the relative performance of PCR and OM may vary among bug ecotopes, eventually showing a greater degree of agreement in domiciles than in peridomestic habitats. Second, the time elapsed between triatomine collection and PCR testing may have allowed some parasite DNA degradation, thus providing a source of discrepancy between techniques; to minimize this, triatomines were frozen at −20 °C until dissection and DNA extraction. Third, the time elapsed since the PCR assays precluded us from running further tests to confirm PCR inhibition in five OM-positive and PCR-negative samples. However, the finding of several other triatomines positive by OM and vkPCR in the same collection sites as the likely inhibited samples further supports the occurrence of potential inhibitory factors. An alternative explanation for this result could be if the examination by OM largely diminished the amount of parasites left for PCR diagnosis, especially if parasite loads were very low. This hypothesis is unlikely given the high sensitivity of conventional PCR [26] relative to the expected density of *T. cruzi* per infected *T. infestans* [20] in an endemic area under active vector-borne transmission [9, 23]. Last, to explicitly account for false-negative and false-positive results, unambiguous determination of *T. cruzi* infection is needed. A larger sample of triatomines collected at relevant ecotopes and tested by both techniques is necessary to implement this analysis.

## Conclusions

Vector infection prevalence is a key component of vectorial capacity and domestic transmission risk. This is the first study that uses site-occupancy models in *T. infestans*. Our study provides a synthesis on the different sources (both biological and technical) of variation of the outcome of OM and PCR-based diagnosis of *T. cruzi* infection in triatomines and identifies research needs for diagnostic improvement.

## Supplementary Information

Additional File 1: Table S1. Chronologically ordered studies comparing OM and PCR diagnostic results of *Trypanosoma cruzi* infection in triatomines.

Additional File 2: Table S2. Database.

## Data Availability

Data are provided within the manuscript or supplementary information files.

## Abbreviations

DNA: Deoxyribonucleic acid
LAMP: Loop-mediated isothermal amplification
OM: Optical microscopy
PCR: Polymerase chain reaction

## References

[1] Abad-Franch F, Ferraz G, Campos C, Palomeque FS, Grijalva MJ, Aguilar HM, et al. Modeling disease vector occurrence when detection is imperfect: infestation of Amazonian palm trees by triatomine bugs at three spatial scales. PLoS Negl Trop Dis. 2010;4:e620. 10.1371/journal.pntd.0000620. 20209149 10.1371/journal.pntd.0000620 PMC2830460

[2] Abad-Franch F, Gurgel-Gonçalves R. The ecology and natural history of wild Triatominae in the Americas. In: Guarneri AA, Lorenzo MG, editors. Triatominae: the biology of chagas disease vectors Entomology in focus, vol. 5. Cham: Springer; 2021.

[3] Bautista-Lopez N, Ndao M. Usefulness of polymerase chain reaction tests in Chagas disease studies. Front Parasitol. 2024;3:1292143. 10.3389/fpara.2024.1292143. 39817172 10.3389/fpara.2024.1292143 PMC11731798

[4] Botto-Mahan C, Ortiz S, Rozas M, Cattan PE, Solari A. DNA evidence of *Trypanosoma cruzi* in the Chilean wild vector *Mepraia spinolai* (Hemiptera: Reduviidae). Mem Inst Oswaldo Cruz. 2005;100:237–9. 10.1590/s0074-02762005000300003. 16113860 10.1590/s0074-02762005000300003

[5] Braz LM, Raiz R Jr, Amato Neto V, Alárcon RS, Gakyia E, Okay TS. The detection of *Trypanosoma cruzi* in *Triatoma infestans*: comparison of a PCR-based assay with microscopical examination (Erratum in: Ann Trop Med Parasitol: 101:498. Amato Neto, V [corrected to Neto, Amato]). Ann Trop Med Parasitol. 2007;101:461–5. 10.1179/136485907X176535. 17550653 10.1179/136485907X176535

[6] Brenière SF, Aznar C, Hontebeyrie M. Vector transmission. In: Telleria J, Tibayrenc M, editors. American Trypanosomiasis Chagas disease one hundred years of research. London: Elsevier; 2017. p. 848.

[7] Brenière SF, Bosseno MF, Telleria J, Carrasco R, Vargas F, Yaksic N, et al. Field application of polymerase chain reaction diagnosis and strain typing of *Trypanosoma cruzi* in Bolivian triatomines. Am J Trop Med Hyg. 1995;53:179–84. 10.4269/ajtmh.1995.53.179. 7677221 10.4269/ajtmh.1995.53.179

[8] Browne A, Guerra C, Alves R, Maia da Costa V, Wilson AL, Pigott DM, et al. The contemporary distribution of *Trypanosoma cruzi* infection in humans, alternative hosts and vectors. Sci Data. 2017;4:170050. 10.1038/sdata.2017.50. 28398292 10.1038/sdata.2017.50 PMC5387921

[9] Cardinal MV, Enriquez GF, Macchiaverna NP, Argibay HD, Fernández MdP, Alvedro A, et al. Long-term impact of a ten-year intervention program on human and canine *Trypanosoma cruzi* infection in the Argentine Chaco. PLoS Negl Trop Dis. 2021;15:e0009389. 10.1371/journal.pntd.0009389. 33979344 10.1371/journal.pntd.0009389 PMC8115854

[10] Cardinal MV, Lauricella MA, Marcet PL, Orozco MM, Kitron U, Gürtler RE. Impact of community-based vector control on house infestation and *Trypanosoma cruzi* infection in *Triatoma infestans*, dogs and cats in the Argentine Chaco. Acta Trop. 2007;103:201–11. 17686448 10.1016/j.actatropica.2007.06.007 PMC2931801

[11] Cardinal MV, Orozco MM, Enriquez GF, Ceballos LA, Gaspe MS, Alvarado-Otegui JA, et al. Heterogeneities in the ecoepidemiology of *Trypanosoma cruzi* infection in rural communities of the Argentinean Chaco. Am J Trop Med Hyg. 2014;90:1063–73. 10.4269/ajtmh.13-0251. 24732461 10.4269/ajtmh.13-0251 PMC4047730

[12] Cardinal MV, Sartor PA, Gaspe MS, Enriquez GF, Colaianni I, Gürtler RE. High levels of human infection with *Trypanosoma cruzi* associated with the domestic density of infected vectors and hosts in a rural area of northeastern Argentina. Parasit Vectors. 2018;11:492. 10.1186/s13071-018-3069-0. 30165892 10.1186/s13071-018-3069-0 PMC6118006

[13] Cerisola JA, Rohwedder RW, Del Prado CE. Rendimiento del xenodiagnóstico en la infección chagásica crónica humana, utilizando ninfas de diferentes especias de triatominos. Bol Chile Parasit. 1971;26:57–8. 5002112

[14] Chiurillo MA, Peralta A, Ramirez JL. Comparative study of *Trypanosoma rangeli* and *Trypanosoma cruzi* telomeres. Mol Biochem Parasitol. 2002;120:305–8. 11897137 10.1016/s0166-6851(02)00005-1

[15] Coura JR. The main sceneries of Chagas disease transmission. The vectors, blood and oral transmissions–a comprehensive review. Mem Inst Oswaldo Cruz. 2015;110:277–82. 10.1590/0074-0276140362. 25466622 10.1590/0074-0276140362 PMC4489464

[16] Dias FdA, Guerra B, Vieira LR, Perdomo HD, Gandara ACP, Amaral RJVd, et al. Monitoring of the parasite load in the digestive tract of *Rhodnius prolixus* by combined qPCR analysis and imaging techniques provides new insights into the trypanosome life cycle. PLoS Negl Trop Dis. 2015;9:e0004186. 10.1371/journal.pntd.0004186. 26496442 10.1371/journal.pntd.0004186 PMC4619730

[17] Dorn P, Engelke D, Rodas A, Rosales R, Melgar S, Brahney B, et al. Utility of the polymerase chain reaction in detection of *Trypanosoma cruzi* in Guatemalan Chagas’ disease vectors. Am J Trop Med Hyg. 1999;60:740–5. 10344645 10.4269/ajtmh.1999.60.740

[18] Enriquez GF, Bua J, Orozco MM, Wirth S, Schijman AG, Gürtler RE, et al. High levels of *Trypanosoma cruzi* DNA determined by qPCR and infectiousness to *Triatoma infestans* support dogs and cats are major sources of parasites for domestic transmission. Infect Genet Evol. 2014;25:36–43. 10.1016/j.meegid.2014.04.002. 24732410 10.1016/j.meegid.2014.04.002

[19] Fleiss JL. Statistical methods for rates and proportions. New York: Wiley; 1973.

[20] Giojalas LC, Catalá SS, Asin SN, Gorla DE. Seasonal changes in infectivity of domestic populations of *Triatoma infestans*. Trans R Soc Trop Med and Hyg. 1990;84:439–42. 2124395 10.1016/0035-9203(90)90355-i

[21] Gurevitz JM, Ceballos LA, Gaspe MS, Alvarado-Otegui JA, Enríquez GF, Kitron U, et al. Factors affecting infestation by *Triatoma infestans* in a rural area of the humid Chaco in Argentina: a multi-model inference approach. PLoS Negl Trop Dis. 2011;5:e1349. 10.1371/journal.pntd.0001349. 22028941 10.1371/journal.pntd.0001349 PMC3196485

[22] Gürtler RE, Gaspe MS, Macchiaverna NP, Enriquez GF, Rodríguez-Planes LI, Fernández MdP, et al. The Pampa del Indio project: districtwide quasi-elimination of *Triatoma infestans* after a 9-year intervention program in the Argentine Chaco. PLoS Negl Trop Dis. 2023;17:e0011252. 10.1371/journal.pntd.0011252. 37093886 10.1371/journal.pntd.0011252 PMC10159358

[23] Gürtler RE, Gaspe MS, Macchiaverna NP, Enriquez GF, Rodríguez-Planes LI, Fernández MdP, et al. The Pampa del Indio project: sustained surveillance and insecticide-based control reduced the prevalence and abundance of *Triatoma infestans* infected with *Trypanosoma cruzi* in the Argentine Chaco. Parasit Vectors. 2023;16:258. 10.1186/s13071-023-05861-7. 37528423 10.1186/s13071-023-05861-7 PMC10394798

[24] Haidamak J, Shimada MK, Klisiowicz DR, Reifur L. *Trypanosoma cruzi* vector infection rate is underestimated in some localities in the state of Bahia. Rev Patol Trop. 2016;45:55–65. 10.5216/rpt.v45i1.39979.

[25] Ibáñez-Cervantes G, Martínez-Ibarra A, Nogueda-Torres B, López-Orduña E, Alonso AL, Perea C, et al. Identification by Q-PCR of *Trypanosoma cruzi* lineage and determination of blood meal sources in triatomine gut samples in México. Parasitol Int. 2013;62:36–43. 10.1016/j.parint.2012.09.003. 22995149 10.1016/j.parint.2012.09.003

[26] Kirchhoff LV, Votava JR, Ochs DE, Moser DR. Comparison of PCR and microscopic methods for detecting *Trypanosoma cruzi*. J Clin Microbiol. 1996;34:1171–5. 8727897 10.1128/jcm.34.5.1171-1175.1996 PMC228976

[27] Lardeux F, Aliaga C, Depickère S. Bias due to methods of parasite detection when estimating prevalence of infection of *Triatoma infestans* by *Trypanosoma cruzi*. J Vector Ecol. 2016;41:285–91. 27860015 10.1111/jvec.12224

[28] Larocca L, Stolowicz FG, Vojnov AA, Suarez FC, Salvá L, Meli S, et al. A simplified molecular tool for detecting the Chagas etiological agent using a vector feces sample in field conditions. J Invertebrate Pathol. 2024;206:108161. 10.1016/j.jip.2024.108161. 10.1016/j.jip.2024.108161 38914370

[29] Lima-Neiva V, Toma HK, Abrantes Aguiar LM, Lopes CM, Dias LP, Monte Gonçalves TC, et al. The connection between *Trypanosoma cruzi* transmission cycles by *Triatoma brasiliensis brasiliensis*: a threat to human health in an area susceptible to desertification in the Seridó, Rio Grande do Norte, Brazil. PLoS Negl Trop Dis. 2021;15:e0009919. 10.1371/journal.pntd.0009919. 34752464 10.1371/journal.pntd.0009919 PMC8577756

[30] MacKenzie DI, Nichols JD, Lachman GB, Droege S, Royle A, Langtimm CA. Estimating site occupancy rates when detection probabilities are less than one. Ecology. 2002;83:2248–55.

[31] Macchiaverna NP, Enriquez GF, Bua J, Fernández MP, Sartor PA, Gürtler RE, et al. Human infectiousness and parasite load in chronic patients seropositive for *Trypanosoma cruzi* in a rural area of the Argentine Chaco. Infect Genet Evol. 2020;78:104062. 10.1016/j.meegid.2019.104062. 31683004 10.1016/j.meegid.2019.104062

[32] Maffey L, Cardinal MV, Ordóñez-Krasnowski PC, Lanati LA, Lauricella MA, Schijman AG, et al. Direct molecular identification of Trypanosoma cruzi discrete typing units in domestic and peridomestic *Triatoma infestans* and *Triatoma sordida* from the Argentine Chaco. Parasitology. 2012;139:1570–9. 23036510 10.1017/S0031182012000856 PMC3749237

[33] Marcet PL, Duffy T, Cardinal MV, Burgos JM, Lauricella MA, Levin MJ, et al. PCR-based screening and lineage identification of *Trypanosoma cruzi* directly from faecal samples of triatomine bugs from northwestern Argentina. Parasitology. 2006;132:57–65. 10.1017/S0031182005008772. 16393354 10.1017/S0031182005008772 PMC1853270

[34] Marti GA, Echeverria MG, Susevich ML, Becnel JJ, Pelizza SA, García JJ. Prevalence and distribution of parasites and pathogens of Triatominae from Argentina, with emphasis on *Triatoma infestans* and Triatoma virus TrV. J Invertebr Pathol. 2009;102:233–7. 19660466 10.1016/j.jip.2009.06.010

[35] Minuzzi-Souza TTC, Nitz N, Cuba CAC, Hagström L, Hecht MM, Santana C, et al. Surveillance of vector-borne pathogens under imperfect detection: lessons from Chagas disease risk (mis)measurement. Sci Rep. 2018;8:151. 10.1038/s41598-017-18532-2.Erratum.In:SciRep.2018May4;8(1):7439. 29317702 10.1038/s41598-017-18532-2 PMC5760667

[36] Moreira OC, Verly T, Finamore-Araujo P, Gomes SAO, Lopes CM, de Sousa DM, et al. Development of conventional and real-time multiplex PCR-based assays for estimation of natural infection rates and *Trypanosoma cruzi* load in triatomine vectors. Parasit Vectors. 2017;10:404. 10.1186/s13071-017-2343-x. 28851417 10.1186/s13071-017-2343-x PMC5576278

[37] Panzera F, Ferreiro MJ, Pita S, Calleros L, Pérez R, Basmadjián Y, et al. Evolutionary and dispersal history of Triatoma infestans, main vector of Chagas disease, by chromosomal markers. Infect Genet Evol. 2014;27:105–13. 10.1016/j.meegid.2014.07.006. 25017654 10.1016/j.meegid.2014.07.006

[38] Pineda D, Paredes B, Russomando G, Sánchez Z. Enfermedad de Chagas: Riesgo de transmisión por especies secundarias de triatominos capturados en etapa de vigilancia entomológica en las dos regiones del Paraguay. Mem Inst Investig Cienc Salud. 2022;20:77–84. 10.18004/mem.iics/1812-9528/2022.020.02.77.

[39] Ramsey JM, Gutiérrez-Cabrera AE, Salgado-Ramírez L, Peterson AT, Sánchez-Cordero V, Ibarra-Cerdeña CN. Ecological connectivity of *Trypanosoma cruzi* reservoirs and *Triatoma pallidipennis* hosts in an anthropogenic landscape with endemic Chagas disease. PLoS ONE. 2012;7:e46013. 10.1371/journal.pone.0046013. 23049923 10.1371/journal.pone.0046013 PMC3458814

[40] Rojas ME, Várquez P, Villarreal MF, Velandia C, Vergara L, Morán-Borges YH, et al. Estudio seroepidemiológico y entomológico sobre la enfermedad de Chagas en un área infestada por *Triatoma maculata* (Erichson 1848) en el centro-occidente de Venezuela [An entomological and seroepidemiological study of Chagas’ disease in an area in central-western Venezuela infested with *Triatoma maculata* (Erichson 1848)]. Cad Saude Publica. 2008;24:2323–33. 10.1590/s0102-311x2008001000013. 18949234 10.1590/s0102-311x2008001000013

[41] Rojas-Cortez M, Pinazo MJ, Garcia L, Arteaga M, Uriona L, Gamboa S, et al. *Trypanosoma cruzi*-infected *Panstrongylus geniculatus* and *Rhodnius robustus* adults invade households in the Tropics of Cochabamba region of Bolivia. Parasit Vectors. 2016;9:158. 10.1186/s13071-016-1445-1. 26984679 10.1186/s13071-016-1445-1 PMC4794895

[42] Russomando G, Rojas de Arias A, Almiron M, Figueredo A, Ferreira ME, Morita K. *Trypanosoma cruzi*: polymerase chain reaction-based detection in dried feces of *Triatoma infestans*. Exp Parasitol. 1996;83:62–6. 10.1006/expr.1996.0049. 8654552 10.1006/expr.1996.0049

[43] Schijman AG. Molecular diagnosis of *Trypanosoma cruzi*. Acta Trop. 2018;184:59–66. 10.1016/j.actatropica.2018.02.019. 29476727 10.1016/j.actatropica.2018.02.019

[44] Schijman AG, Lauricella MA, Marcet PL, Duffy T, Cardinal MV, Bisio M, et al. Differential detection of *Blastocrithidia triatomae* and *Trypanosoma cruzi* by amplification of 24s ribosomal RNA genes in faeces of sylvatic triatomine species from rural northwestern Argentina. Acta Trop. 2006;99:50–4. 16887092 10.1016/j.actatropica.2006.06.010

[45] Sergeant, ESG, 2018. Epitools Epidemiological Calculators. Ausvet. http://epitools.ausvet.com.au.

[46] Sherlock IA, Carcavallo RU, Galíndez Girón I. List of natural and experimental flagellate infections in several triatomine species. In: Carcavallo GG, Jurgerg L, editors. Atlas of Chagas disease vectors in the Americas, vol. 1. Brasil: Editora Fiocruz Rio de Janeiro; 1998. p. 289–98.

[47] Shikanai-Yasuda MA, Ochs DE, Tolezano JE, Kirchhoff LV. Use of the polymerase chain reaction for detecting *Trypanosoma cruzi* in triatomine vectors. Trans R Soc Trop Med Hyg. 1996;90:649–51. 9015504 10.1016/s0035-9203(96)90419-8

[48] Skalski JR, Ryding KE, Millspaugh JJ, 2005. Analysis of population indices. In: Skalski JR, Ryding KE, Millspaugh JJ, editors. Wildlife demography. Academic Press, pp 359–433.

[49] Stevens L, Lima-Cordón RA, Helms Cahan S, Dorn PL, Monroy MC, Axen HJ, et al. Catch me if you can: under-detection of *Trypanosoma cruzi* (Kinetoplastea: Trypanosomatida) infections in *Triatoma dimidiata* s.l. (Hemiptera: Reduviidae) from Central America. Acta Trop. 2021;224:106130. 10.1016/j.actatropica.2021.106130. 34536368 10.1016/j.actatropica.2021.106130

[50] Urbano P, Hernández C, Velásquez-Ortiz N, Ballesteros N, Páez-Triana L, Vega L, et al. Transmission ecology of *Trypanosoma cruzi* by *Rhodnius prolixus* (Reduviidae: Triatominae) infesting palm-tree species in the Colombian Orinoco, indicates risks to human populations. PLoS Negl Trop Dis. 2024;18:e0011981. 10.1371/journal.pntd.0011981. 38377140 10.1371/journal.pntd.0011981 PMC10906903

[51] Vallejo GA, Guhl F, Schaub GA. Triatominae-*Trypanosoma cruzi*/*T. rangeli*: vector-parasite interactions. Acta Trop. 2009;110:137–47. 10.1016/j.actatropica.2008.10.001. 18992212 10.1016/j.actatropica.2008.10.001

[52] Vallejo GA, Suárez YO, Jenny L, Gutiérrez SA, Carranza JC. *Trypanosoma rangeli*: un protozoo infectivo y no patógeno para el humano que contribuye al entendimiento de la transmisión vectorial y la infección por *Trypanosoma cruzi*, agente causal de la enfermedad de Chagas. Rev Acad Colomb Cienc Exact Fis Nat. 2015;39: 111–122. http://www.scielo.org.co/scielo.php?script=sci_arttext&pid=S0370-39082015000100011&lng=en&nrm=iso. Accessed 20 Jun 2024.

[53] Velásquez-Ortiz N, Herrera G, Hernández C, Muñoz M, Ramírez JD. Discrete typing units of *Trypanosoma cruzi*: geographical and biological distribution in the Americas. Sci Data. 2022;9:360. 10.1038/s41597-022-01452-w. 35750679 10.1038/s41597-022-01452-w PMC9232490

[54] WHO. 2020. Ending the neglect to attain the sustainable development goals—A road map for neglected tropical diseases 2021–2030. https://www.who.int/publications/i/item/9789240010352. Accessed 14 Jun 2024.

[55] World Health Organization [WHO]. Chagas disease fact sheet. 6 Apr 2023. https://www.who.int/news-room/fact-sheets/detail/chagas-disease-(american-trypanosomiasis). Accessed 18 May 2023.

